# Antioxidant and anti-inflammatory effects of intravenously injected adipose derived mesenchymal stem cells in dogs with acute spinal cord injury

**DOI:** 10.1186/s13287-015-0236-5

**Published:** 2015-11-26

**Authors:** Yongsun Kim, Sung-ho Jo, Wan Hee Kim, Oh-Kyeong Kweon

**Affiliations:** BK21 PLUS Program for Creative Veterinary Science Research, Research Institute for Veterinary Science and College of Veterinary Medicine, Seoul National University, 1 Gwanak-ro, Gwanak-gu, Seoul, 151-742 South Korea

**Keywords:** Antioxidant, Anti-inflammatory, Spinal cord injury, Mesenchymal stem cells, Dog

## Abstract

**Introduction:**

Mesenchymal stem cells can potentially be used in therapy for spinal cord injury (SCI). Methylprednisolone sodium succinate (MPSS) has been used as a scavenging agent in acute SCI treatment, but its use no longer recommended. This study aimed to identify ways to reduce the usage and risk of high doses of glucocorticoid steroids, and determine whether AD-MSCs could be used as an early alternative treatment modality for acute SCI.

**Methods:**

Sixteen adult beagle dogs with SCI were assigned to four treatment groups: control, MPSS, AD-MSCs, and AD-MSCs + MPSS. Additionally, one dog was used to evaluate the distribution of AD-MSCs in the body after injection. AD-MSCs (1 × 10^7^ cells) were injected intravenously once a day for 3 days beginning at 6 hours post-SCI. MPSS was also injected intravenously according to the standard protocol for acute SCI. A revised Tarlov scale was used to evaluate hindlimb functional recovery. The levels of markers for oxidative metabolism (3-nitrotyrosine, 4-hydroxynonenal, and protein carbonyl) and inflammation (cyclooxygenase-2, interleukin-6, and tumor necrosis factor-α) were also measured.

**Results:**

At 7 days post-treatment, hindlimb movement had improved in the AD-MSCs and AD-MSCs + MPSS groups; however, subjects in the groups treated with MPSS exhibited gastrointestinal hemorrhages. Hematoxylin and eosin staining revealed fewer hemorrhages and lesser microglial infiltration in the AD-MSCs group. The green fluorescent protein-expressing AD-MSCs were clearly detected in the lung, spleen, and injured spinal cord; however, these cells were not detected in the liver and un-injured spinal cord. Levels of 3-nitrotyrosine were decreased in the MPSS and AD-MSCs + MPSS groups; 4-hydroxynenonal and cyclooxygenase-2 levels were decreased in all treatment groups; and interleukin-6, tumor necrosis factor-α, and phosphorylated-signal transducer and activator transcription 3 levels were decreased in the AD-MSCs and AD-MSCs + MPSS groups.

**Conclusion:**

Our results suggest that early intravenous injection of AD-MSCs after acute SCI may prevent further damage through enhancement of antioxidative and anti-inflammatory mechanisms, without inducing adverse effects. Additionally, this treatment could also be used as an alternative intravenous treatment modality for acute SCI.

## Introduction

Spinal cord injury (SCI) results from the primary mechanical damage that occurs when structural thresholds are surpassed, which in turn leads to immediate physical and biochemical alterations. These alterations are followed by a secondary injury mechanism that includes a variety of vascular, biochemical, and cellular processes [1]. SCI is generally followed by immediate hematoma formation and oxidative and inflammatory responses. These events involve enzyme activation, mediator release, inflammatory cell migration, glial activation, and neuronal tissue degradation [2, 3]. Hemorrhages cause the extracellular spaces in the spinal cord to become exposed to hemoglobin and its breakdown products, which subsequently results in free radical generation. Infiltrating neutrophils can directly damage tissue by producing reactive oxygen species and secreting proinflammatory mediators [4]. Moreover, microglial activation is involved in clearing the hematoma, and activated microglia secrete a variety of cytokines. Following SCI, the progression of these events inhibits axonal regeneration in the central nervous system, such that patients are unable to recover from the neurological damage.

The neuroprotective agent methylprednisolone sodium succinate (MPSS) has been widely used in the clinical treatment of acute SCI [5, 6]. However, recent retrospective cohort studies have demonstrated the lack of a statistically significant difference between clinical outcomes in SCI patients treated with and without MPSS [7]. The therapeutic effect of MPSS on SCI therefore remains controversial. As the current standard effective therapeutic agent for the clinical treatment of acute SCI, MPSS has been shown to alleviate secondary injury by decreasing inflammation and spinal cord ischemia, as well as by inhibiting cell membrane lipid peroxidation (LP). However, administration of high doses of glucocorticoid steroids causes many complications, including increased incidence of infection, pneumonia, pressure sores, gastrointestinal bleeding, and deep vein thrombosis [8].

Much of the current research on SCI is focused on developing treatment methods that limit or prevent secondary injury. Minimizing secondary injury is generally achieved by ensuring adequate perfusion and oxygenation and by ancillary administration of neuroprotective agents for improving clinical signs. Transplantation of mesenchymal stem cells (MSCs) has been shown to provide neuroprotection, increase neuronal regeneration, and ameliorate the clinical signs of SCI [9, 10]. In these studies, the transplanted MSCs not only improved the inflammatory environment and enhanced the survival of endogenous nerve cells, but also reduced fibrosis formation and were able to partially differentiate into neural cells [9, 11].

We hypothesized that adipose-derived MSCs (AD-MSCs) intravenously transplanted into animals with acute SCI would alleviate inflammation at the injured site because of their antioxidant and anti-inflammatory properties. Based on our hypothesis, administration of AD-MSCs should ultimately result in less scar tissue formation, aid ingrowth of neural progenitor cells, and improve limb function. The present study was conducted to reduce the usage and risk of high doses of glucocorticoid steroids, and to determine whether AD-MSCs could be used as an early alternative treatment modality for acute SCI.

## Methods

### Isolation and culture of canine AD-MSCs

MSCs derived from the adipose tissue of canine hip fat were isolated and characterized [11]. Specifically, adipose tissue was collected aseptically from the subcutaneous fat of a 2-year-old beagle dog under anesthesia. Tissues were washed with phosphate-buffered saline (PBS), minced, and digested with collagenase type I (1 mg/ml; Sigma-Aldrich, St. Louis, MO, USA) at 37 °C for 30–60 minutes with intermittent shaking. The suspension was filtered through a 100 μm nylon mesh and centrifuged to separate floating adipocytes from stromal cells. Preadipocytes in the stromal vascular fraction were plated at 8000–10,000 cells/cm^2^ in T175 culture flasks containing Dulbecco’s modified Eagle’s medium (DMEM; Gibco-BRL, Grand Island, NY, USA) supplemented with 3.7 g/l sodium bicarbonate, 1 % penicillin and streptomycin, 1.7 mM l-glutamine, 0.1 mM β-mercaptoethanol, and 10 % fetal bovine serum (FBS). The cells were incubated in a humidified atmosphere at 37 °C with 5 % CO_2_. Unattached cells and residual nonadherent red blood cells were removed after 24 hours using a PBS wash, and the cell medium was replaced every 2 days. At passage 3, the cells were used for the following experiments.

### Animals

Seventeen healthy 2–3-year-old beagle dogs weighing 8.5 ± 2.2 kg were used in the study. All dogs were clinically judged to be in good health and to have a normal neurological status. During the experiment, all dogs were cared for in accordance with the animal care guidelines of the Institute of Laboratory Animal Resources at Seoul National University, Korea. Sixteen dogs were assigned to four groups based on the treatments: control (no treatment after SCI; *n* = 4), MPSS (administration of MPSS after SCI; *n* = 4), AD-MSCs (administration of AD-MSCs; *n* = 4), and AD-MSCs + MPSS (administration of both AD-MSCs and MPSS; *n* = 4). One additional dog was used to evaluate distribution of MSCs in the body after injection. The Institutional Animal Care and Use Committee of Seoul National University approved the experimental design (SNU-111102-8).

### Induction of SCI

SCI was induced using a previously described balloon compression method [10]. Briefly, the dogs were medicated and anesthetized with tramadol (4 mg/kg intravenously, Toranzin; Samsung Pharm. Ind. Co., Seoul, Korea), propofol (6 mg/kg intravenously, Provive 1 %; Claris Lifesciences, Ahmedabad, India), and atropine sulfate (0.05 mg/kg subcutaneously, Atropine; Jeil Pharm., Yongin, Korea). Anesthesia was maintained with isoflurane (Forane solution; Choongwae Pharm. Co., Seoul, Korea) at a minimum alveolar concentration (MAC) of 1.5 throughout the procedure. Electrocardiography, pulse oximetry, respiratory gas analysis, and rectal temperature measurement were performed using an anesthetic monitoring system (Datex-Ohmeda S/5; GE Healthcare, Little Chalfont, UK). The dogs were suspended in a ventral recumbent position, and hemilaminectomy was performed through a left paramedian approach at the fourth lumbar segment (L4). A hole of 3–5 mm was made in the left vertebral arch at L4 using a high-speed pneumatic burr, and a 4-French embolectomy catheter (Edwards Lifesciences, Irvine, CA, USA) was inserted into the hole. Under fluoroscopic guidance, the balloon catheter was advanced until the tip was positioned at the cranial margin of the first lumbar segment (L1) vertebral body. The balloon was then inflated by injecting 50 μl/kg contrast agent (Omnipaque; GE Healthcare) diluted with saline in a 50:50 proportion. The balloon’s positioning was confirmed using fluoroscopy. According to a previous study [9], this SCI model occludes more than 85 % of the spinal canal, as confirmed by computed tomography. Following induction of the injury, the soft tissue and skin were closed using standard methods. The balloon was fixed with a Chinese finger-type suture and removed after 6 hours. After the operation, the dogs were monitored in an ICU, and manual bladder expression was performed at least three times daily if needed.

### Administration of MPSS and AD-MSCs

Administration of MPSS and AD-MSCs was initiated following removal of the embolectomy catheter. MPSS (30 mg/kg, Methysol; Kunhwa Pharmaceutical Co., Seoul, Korea) was bolus injected into the cephalic vein and followed by continuous rate infusion (5.4 mg/kg/hour) for the next 47 hours [6]. A suspension of 1 × 10^7^ allogenic AD-MSCs in 10 ml lactated Ringer’s solution was administered intravenously once a day for 3 successive days.

### Labeling and tracking of AD-MSCs

Twenty-four hours before transfection, 4 × 10^6^ HEK293 cells were seeded into a 100 mm dish. The following day, a lentiviral packaging mix (System Biosciences, San Diego, CA, USA) encoding viral proteins Gag-Pol, Rev, and VSV-G and lentiviral transgene plasmids were transfected into each well for lentivirus production using Turbofect (Thermo Scientific, Waltham, MA, USA). Green fluorescent protein (GFP)-expressing virus particles were collected and transduced into AD-MSCs at passage 1. After the AD-MSCs reached 90 % confluence, the cells were selected by puromycin (3 μg/ml; Gibco-BRL). Approximately 30 % of cells were successfully transduced after puromycin selection. The AD-MSCs were subcultured, and passage 3 cells were used for the following experiments. The GFP-labeled AD-MSCs were intravenously injected after SCI as already described. The dog was sacrificed at 7 days after transplantation and tissue samples from the lung, liver, spleen, normal spinal cord, and injured spinal cord were collected. GFP protein was identified in each specimen using western blot analysis. The injured spinal cord was fixed in a 10 % formalin solution and embedded in paraffin. Longitudinal sections were made and the tissue was stained with 4′,6-diamidino-2-phenylindole (DAPI, 1:100; Sigma-Aldrich) to identify nuclei. Slides were observed using a fluorescent microscope.

### Clinical assessment

Behavioral assessments were performed on days 2, 4, and 7 after the operation to evaluate functional recovery of the hind limbs. Each dog was videotaped from both sides and behind during the neurologic examination. Using video footage and the revised Tarlov scale [12], the dogs’ gaits were scored independently by two individuals blinded to the experimental condition. The animal’s vital signs and operation site were also checked. A fecal occult blood test (Cell Biolabs, Inc., San Diego, CA, USA) was performed to evaluate any adverse gastrointestinal effects of intravenous administration of AD-MSCs and/or MPSS.

### Histopathological assessment

The dogs were sacrificed through intravenous injection of potassium chloride under general anesthesia at 7 days after transplantation. The spinal cord from the 12th thoracic segment (T12) to the third lumbar segment (L3) was extracted by dissection. Each section was then placed in 10 % sucrose/PBS at 4 °C for 12 hours and subsequently immersed in 20 % sucrose solution overnight at 4 °C. The sample was divided into two parts longitudinally. One-half of each section was immediately frozen with liquid nitrogen for western blot analysis and enzyme-linked immunosorbent assay (ELISA) tests. The other half was embedded in optimal cutting temperature compound (Surgipath®; Leica Biosystems Richmond, Inc., Richmond, IL, USA), frozen, and cut longitudinally into 12 μm sections with a cryomicrotome. These sections were mounted on silane-coated glass slides and stained with hematoxylin and eosin (H&E) or Luxol fast blue stain. The hemorrhagic area was measured by tracing the hemorrhagic margin and calculated using an image analysis program (ImageJ; NIH, Bethesda, MD, USA). The number of microglia was counted manually in the high-powered field from five randomly selected areas of the injured area margin.

### Oxidant metabolite assessment

The peroxynitrite (PN) formation of 3-nitrotyrosine (3-NT), the LP product 4-hydroxynenonal (4-HNE), and the protein oxidation-derived protein carbonyls (PC) were used as markers for oxidative damage. The level of each oxidation product (3-NT, 4-HNE, and PC) was assessed using ELISA kits (Cell Biolabs, Inc.). The samples were placed into the wells of the 3-NT, 4-HNE, and PC conjugate-coated plates and incubated at room temperature for 10 minutes. After incubation, the secondary antibody was added to each of the wells, incubated at room temperature for 1 hour, washed three times with washing buffer, and incubated with secondary antibody–horseradish peroxidase conjugate for 1 hour. The wells were washed, the substrate solution added for color change, and absorbance measured at 450 nm.

### Western blot analysis

The organ tissues and the frozen half of each injured spinal cord specimen were used for western blot analysis. Briefly, the tissue was washed twice with PBS, and then homogenized with a sonicator in lysis buffer (20 mM Tris at pH 7.5, 1 mM ethylenediaminetetraacetic acid, 1 mM ethylene glycol tetraacetic acid, 1 % Triton X-100, 1 mg/ml aprotinin, 1 mM phenylmethylsulfonylfluoride, 0.5 mM sodium orthovanadate) on ice for 30 minutes. Lysates were cleared by centrifugation (10 minutes at 15,000 rpm, 4 °C), and protein concentrations were determined using the Bradford method [13]. Equal amounts of protein (20 μg) were resolved by electrophoresis on 10 % sodium dodecyl sulfate–polyacrylamide gels and transferred to polyvinylidene fluoride membranes. Membrane blots were washed with TBST (10 mM Tris–HCl, pH 7.6, 150 mM NaCl, 0.05 % Tween-20), blocked with 5 % skimmed milk for 1 hour, and incubated with the appropriate primary antibodies at the recommended dilutions. The antibodies used included antibodies against actin (A3853; Sigma-Aldrich), GFP (MA515256; Thermo Scientific), interleukin (IL)-6 (ab6672; Abcam, Cambridge, UK), and cyclooxygenase-2 (COX-2, sc-7951), tumor necrosis factor alpha (TNFα, sc-1350), phosphorylated signal transducer and activator of transcription 3 (pSTAT3, sc-8001-R), β3-tubulin (sc-69966), glial fibrillary acidic protein (GFAP, sc-65343), and galactosylceramidase (GalC, sc-67352) (Santa Cruz Biotechnology, Santa Cruz, CA, USA). The primary antibodies (1:1000) were diluted in TBST. The membrane was then washed, and the primary antibodies were detected with goat anti-rabbit IgG or goat anti-mouse IgG conjugated to horseradish peroxidase (1:5000; Invitrogen, Waltham, MA, USA). Bands were visualized using enhanced chemiluminescence (Invitrogen).

### Statistical analysis

All results are expressed as mean ± standard deviation (SD). Statistical analysis was performed using a commercially available statistical software program (SPSS Statistics, version 21.0; IBM Corp., Armonk, NY, USA). In all experiments, Kruskal–Wallis tests were followed by Mann–Whitney U tests to compare between groups. *P* <0.05 was considered significant.

## Results

### Clinical assessment

**Fig. 1:**
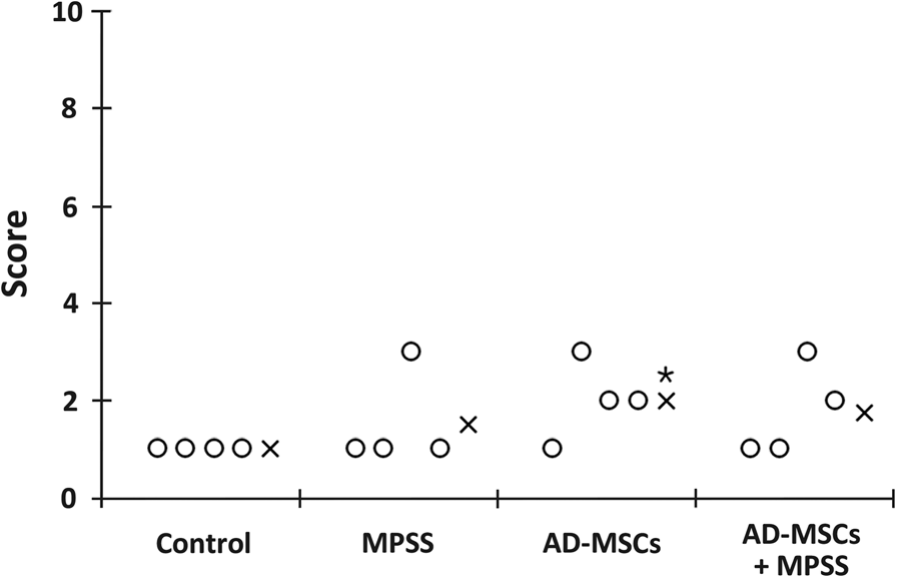
Revised Tarlov scores. Motor function outcome at 7 days after MPSS and AD-MSCs administration. The AD-MSCs group had significantly enhanced motor function compared with the control group at 7 days post treatment. *×* mean. *P <0.05 compared with the control group. *AD-MSCs* adipose-derived mesenchymal stem cells, *MPSS* methylprednisolone sodium succinate

All experimental dogs had no movement and muscle tone in the hind limbs after SCI. However, subjects in the AD-MSCs group had significantly enhanced motor function compared with those in the control group at 7 days post treatment (*P* <0.05; Fig. 1, Table 1). In addition, gastrointestinal hemorrhage was not observed in the control and the AD-MSCs groups but was observed in the MPSS and AD-MSCs + MPSS groups until 4 days post SCI (Table 2). Additionally, one in four dogs in the AD-MSCs + MPSS group and one in three dogs in the MPSS group exhibited gastrointestinal hemorrhages at 4 days post SCI. Other adverse effects such as wound infections or delayed healing were not observed in any of the treatment groups.

**Table 1:** Revised Tarlov scores

Group	*n*	Mean	Standard deviation	*P* value
Control	4	1.00	0	0.317
MPSS	4	1.50	1.00	
Control	4	1.00	0	0.046
AD-MSCs	4	2.00	0.82	
Control	4	1.00	0	0.131
AD-MSCs + MPSS	4	1.75	0.96	
MPSS	4	1.50	1.00	0.350
AD-MSCs	4	2.00	0.82	
MPSS	4	1.50	1.00	0.617
AD-MSCs + MPSS	4	1.75	0.96	
AD-MSCs	4	2.00	0.82	0.647
AD-MSCs + MPSS	4	1.75	0.96	

*P* values were calculated using the Mann–Whitney U test

*AD-MSCs* adipose-derived mesenchymal stem cells, *MPSS* methylprednisolone sodium succinate

**Table 2:** Percentage of gastrointestinal hemorrhage

Group	Time after treatment		
	2 days	4 days	7 days
Control	0 % (0/4)	0 % (0/4)	0 % (0/4)
MPSS	100 % (4/4)	75 % (3/4)	0 % (0/4)
AD-MSCs	0 % (0/4)	0 % (0/4)	0 % (0/4)
AD-MSCs + MPSS	100 % (4/4)	25 % (1/4)	0 % (0/4)

*AD-MSCs* adipose-derived mesenchymal stem cells, *MPSS* methylprednisolone sodium succinate

### Distribution of AD-MSCs

**Fig. 2:**
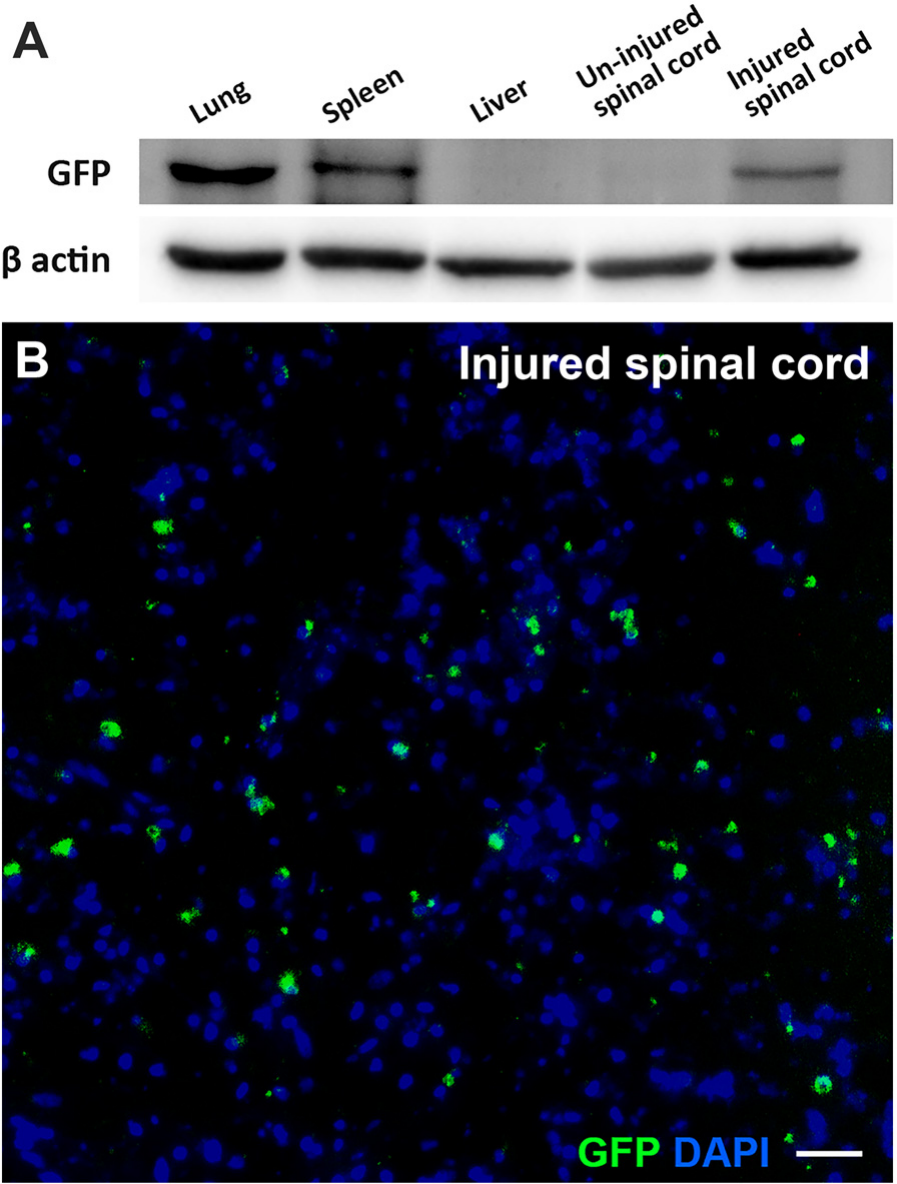
GFP distribution at 7 days after treatment. **a** GFP was detected in the lung, spleen, and injured spinal cord at 7 days after intravenous administration of AD-MSCs. However, GFP was not detected in the liver and uninjured normal spinal cord. **b** GFP-labeled AD-MSCs were observed in the epicenter of the injured spinal cord. Bar: 50 μm. *DAPI* 4′,6-diamidino-2-phenylindole, *GFP* green fluorescent protein

To determine the engraftment of intravenously injected AD-MSCs, GFP was examined at 7 days after administration of GFP-expressing AD-MSCs. GFP was detected in the lung, spleen, and injured spinal cord, but was not detected in the liver and uninjured spinal cord (Fig. 2a). The expression of GFP in the lung was relatively higher than other organ tissues. GFP-labeled AD-MSCs were observed in the epicenter of the injured spinal cord (Fig. 2b).

### Histopathological assessment

**Fig. 3:**
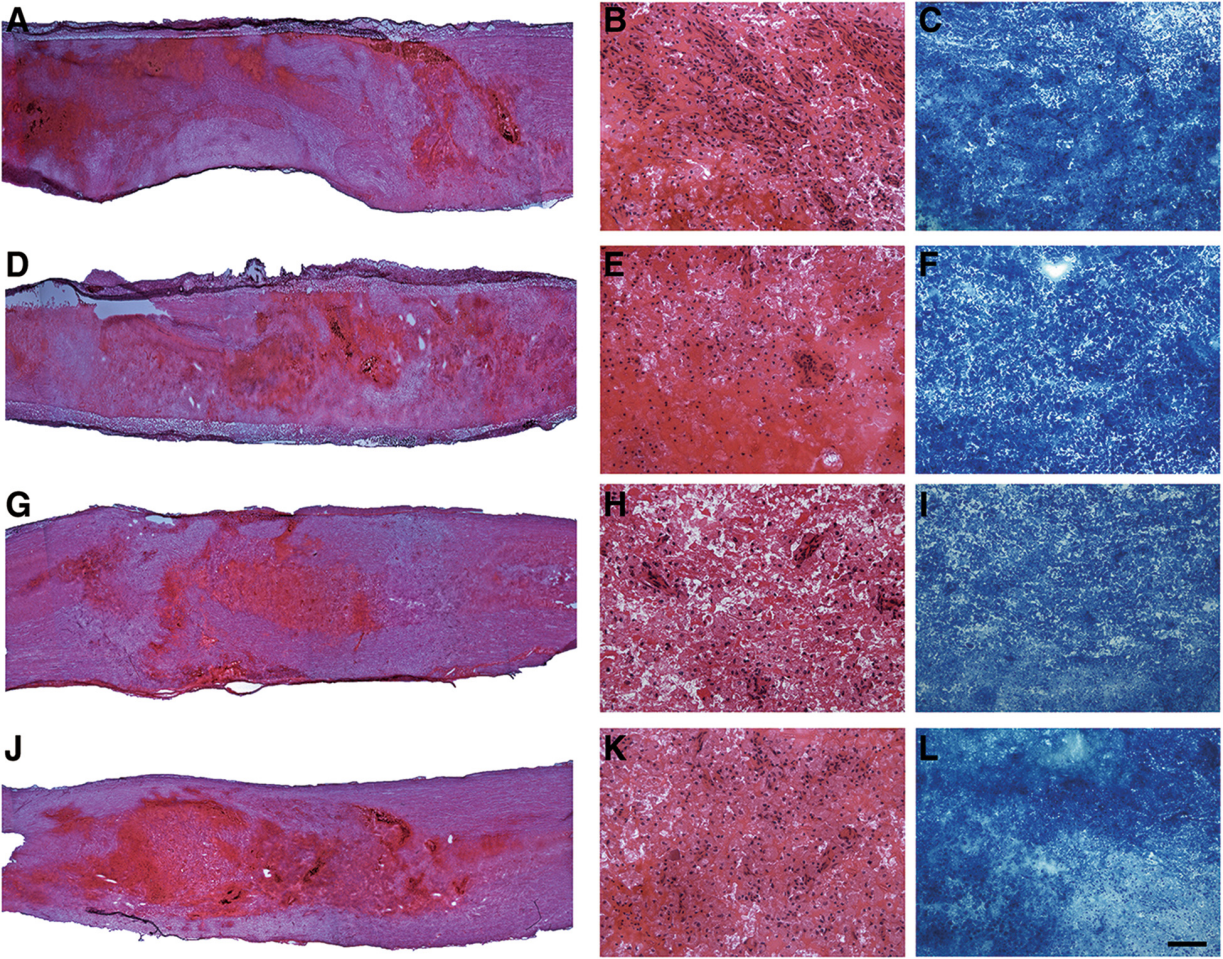
Histologic analysis of spinal cord lesions stained with H&E and Luxol fast blue. Control group **a**–**c**, MPSS group **d**–**f**, AD-MSCs group **g**–**i**, and AD-MSCs + MPSS group **j**–**l** were observed. In the H&E staining **a**, **d**, **g**, **j**, all groups showed severe hemorrhage and infiltration of microglial cells in the injured part of the spinal cord. The injured parenchyma of spinal cord was composed of demyelinated neurons, cell debris, mild fibrosis, and hemorrhage in the high-powered field **b**, **e**, **h**, **k**. The AD-MSCs group showed less hemorrhaging and fewer inflammatory responses compared with other groups. In addition, all groups exhibited severe demyelination of nerve fibers in the SCI lesion stained with Luxol fast blue **c**, **f**, **i**, **l**. Bar: **a**, **d**, **g**, **j** 200 μm; **b**, **c**, **e**, **f**, **h**, **i**, **k**, **l** 25 μm

**Fig. 4:**
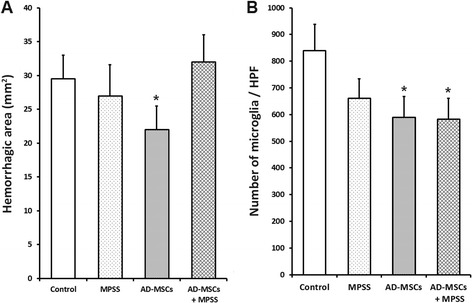
**a** The hemorrhagic area was decreased in the AD-MSCs group compared with other groups. **b** The infiltration of microglia was decreased in the AD-MSCs and AD-MSCs + MPSS groups. Data presented as mean ± SD. *P <0.05 compared with the control group. *AD-MSCs* adipose-derived mesenchymal stem cells, *HPF* high-powered field, *MPSS* methylprednisolone sodium succinate

Seven days after the initial transplantation, the average damaged lesion size per section of groups was 68.4 ± 4.5 mm^2^. There were no significant differences in the area of the lesion among the four groups (Fig. 3). In the low-powered field, H&E-stained sections from all groups showed severe hemorrhage and infiltration of microglial cells in the injured part of the spinal cord. In the high-powered field, an injured spinal cord parenchyma composed of demyelinated neurons, cell debris, and mild fibrosis as well as hemorrhage was observed. Luxol fast blue staining also showed demyelination in the injured area. However, hemorrhages were less commonly observed in the AD-MSCs group and a lower inflammatory response was observed in the AD-MSCs and AD-MSCs + MPSS groups (Fig. 4).

### Antioxidant effects

**Fig. 5:**
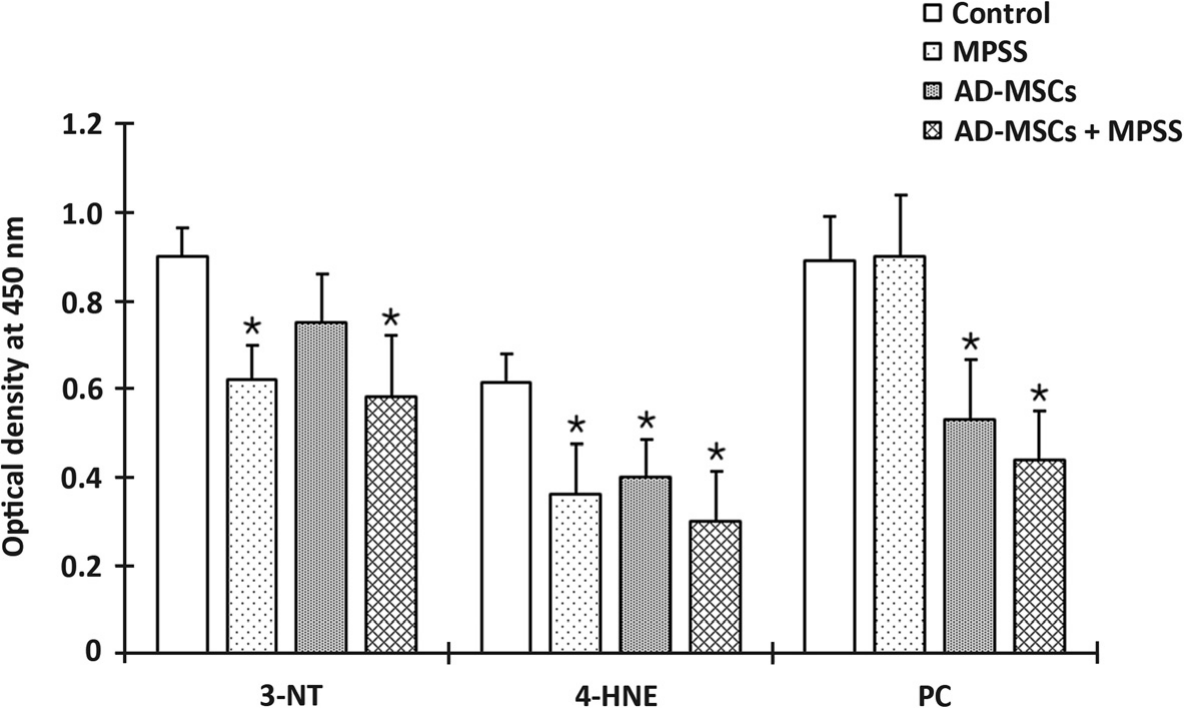
Oxidant metabolite levels (3-NT, 4-HNE, and PC) at 7 days after treatment. The level of 3-NT was decreased in the MPSS and AD-MSCs + MPSS groups compared with the other groups. The level of 4-HNE was decreased in all treatment groups compared with the control group. In addition, the level of PC was decreased in the AD-MSCs and AD-MSCs + MPSS groups when compared with the other two groups. Data presented as mean ± SD. *P <0.05 compared to the control group. *AD-MSCs* adipose-derived mesenchymal stem cells , *4-HNE* 4-hydroxynenonal, *MPSS* methylprednisolone sodium succinate, *3-NT* 3-nitrotyrosine, *PC* protein carbonyls

The level of 3-NT (a PN marker) was significantly decreased in the MPSS and AD-MSCs + MPSS groups compared with other groups (*P* <0.05; Fig. 5). The level of 4-HNE (an LP product) was significantly decreased in all treatment groups compared with the control group (*P* <0.05). In addition, the level of PC (a protein oxidation-related product) was significantly decreased in the AD-MSCs and AD-MSCs + MPSS groups compared with the other two groups (*P* <0.05).

### Anti-inflammatory effects

**Fig. 6:**
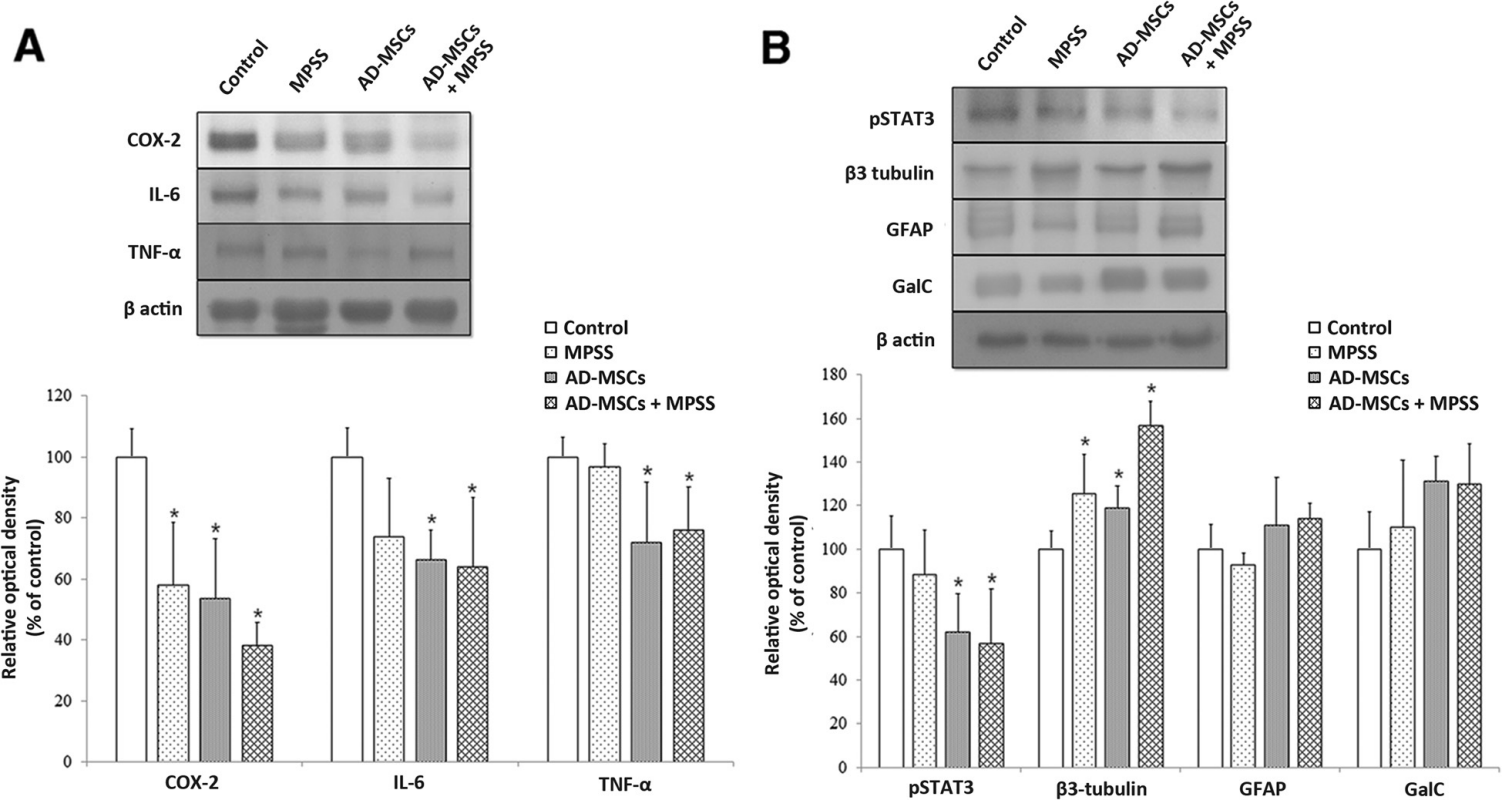
Inflammation and astrogliosis at 7 days after treatment. **a** Inflammatory markers (COX-2, IL-6, TNF-α). COX-2 levels were decreased in all treatment groups. The levels of IL-6 and TNFα were decreased in AD-MSCs and AD-MSCs + MPSS groups. **b** Astrogliosis and neuronal markers (pSTAT3, β3-tubulin, GFAP, GalC). Expression of pSTAT3 was decreased in the AD-MSCs and AD-MSCs + MPSS groups. β3-tubulin levels were increased in all treatment groups. Data presented as mean ± SD. *P <0.05 compared with the control group. *AD-MSCs* adipose-derived mesenchymal stem cells, *COX* cyclooxygenase, *GalC* galactosylceramidase, *GFAP* glial fibrillary acidic protein, *IL* interleukin, *MPSS* methylprednisolone sodium succinate, *pSTAT3* phosphorylated signal transducer and activator of transcription 3, *TNFα* tumor necrosis factor alpha

A decrease in the level of COX-2 was observed in the MPSS group (*P* <0.05); however, IL-6 and TNFα levels were not significantly different from that of the control group (Fig. 6a). On the other hand, the levels of COX-2, IL-6, and TNFα were significantly decreased in the AD-MSCs and AD-MSCs + MPSS groups when compared with those in the control group (*P* <0.05).

### Astrogliosis and neuronal cells

The expression of pSTAT3 was significantly decreased in the AD-MSCs and AD-MSCs + MPSS groups when compared with the control group (*P* <0.05; Fig. 6b). In addition, β3-tubulin levels were increased in all treatment groups as compared with the control group (*P* <0.05). The levels of GFAP, a marker of reactive astrocytes, and GalC, a marker of mature oligodendrocytes, were increased in the AD-MSCs and AD-MSCs + MPSS groups when compared with those in the control group.

## Discussion

The objective of this study was to investigate the antioxidant and anti-inflammatory effects of intravenously injected AD-MSCs in an acute SCI model and to compare these effects with those of MPSS, which is used as a neuroprotective agent for the treatment of acute SCI. Administration of AD-MSCs improved hind-limb functions and reduced the occurrence of adverse effects that are associated with high doses of glucocorticoid steroids. MPSS treatment also resulted in some beneficial effects on the clinical signs, but resulted in the adverse effect of gastrointestinal hemorrhage caused by peptic ulceration. This complication may develop because MPSS inhibits phospholipase A_2_ action, which converts arachidonic acid to COX. These actions of MPSS lead to nonselective inhibition of COX-1 and COX-2. The COX-2 enzyme is involved in inflammatory responses, especially prostaglandin E_2_, but COX-1 is thought to only be involved in normal physiological functions, such as gastrointestinal mucous production, kidney water excretion, and platelet activation [14]. However, AD-MSCs administration did not produce this adverse effect, which suggests that MSCs selectively inhibit COX-2, but not COX-1. It is therefore important to minimize gastrointestinal effects, since they can result in fatal conditions such as gastrointestinal perforation, anemia, secondary infection, and sepsis if left untreated [15, 16].

In the present study, administration of MPSS inhibited nitration and LP, and AD-MSCs inhibited LP and protein oxidation. These antioxidant effects of MPSS correspond well with those reported previously [17]. The effects of MPSS were due to a membrane stabilizing action that inhibits LP by limiting the fluidity of the phospholipids in the neural cell membranes, thereby stunting the LP chain reaction. The MSCs exhibited antioxidant activity through LP prevention, increasing levels of glutathione and superoxide dismutase, and modulating the pathways of antioxidant-related protein activation [18]. Moreover, the histopathological findings in the present study showed that AD-MSCs transplantation reduced intraparenchymal hemorrhage, reduced migration of microglia, and decreased the expression of oxidative metabolites. The reduced hemorrhaging caused by AD-MSCs administration might directly reduce the release of free radicals from hematomas in addition to reducing microglial activation. In this study, AD-MSCs and MPSS both showed antioxidant effects, but appeared to do so by different mechanisms. Accordingly, a combination therapy of AD-MSCs and MPSS for acute SCI was required for optimal antioxidation with fewer adverse effects.

In the present study, intravenous injection of AD-MSCs decreased levels of inflammatory cytokines including COX-2, IL-6, and TNFα, which may have resulted from the inhibition of microglial activation and inflammatory responses. However, MPSS does not decrease levels of IL-6 and TNFα, and it seems that high doses of MPSS exert greater antioxidant effect than anti-inflammatory effects [19]. In addition, the unclear neurological improvement and anti-inflammatory effects of MPSS in the present study might support the results indicating a low therapeutic effect. Previous studies have reported that MSCs exert immunomodulatory effects by attenuating and modulating excessive inflammatory reactions [20, 21]. MSCs have been shown to reduce levels of proinflammatory cytokines such as interferon gamma, TNFα, and IL-6 and to increase expression of indoleamine 2,3-dioxygenase, which suppresses T-cell responses and promotes immunological tolerance [22]. In spinal microglia, IL-6 is associated with inflammatory cytokine signaling to induce STAT3, and also has the ability to trigger reactive astrogliosis [23, 24]. In the present study, AD-MSCs decreased levels of IL-6 which is related to pSTAT3 and astrogliosis, suggesting an anti-astrogliosis effect. Reactive astrocytes contribute to glial scar formation and inhibition of axonal outgrowth. According to recent studies, however, reactive astrocytes also provide beneficial effects that protect adjacent neural tissue and secrete growth-promoting neurotrophic factors [25, 26]. The existing GFAP-expressing reactive astrocytes could create the neuroprotective environment for neurogenesis with transplanted cells.

MSCs transplantation strategies require a safe and efficient method of cellular delivery. In animal models of SCI, the most common delivery method is direct injection into the injured site, which allows many cells to be transplanted effectively, albeit through an extremely invasive procedure that may lead to further injuries. Consequently, this method may be very difficult to implement in human subjects. Therefore, less invasive methods for cell delivery have been investigated and intravenous administration has been identified as an ideal and preferable minimally invasive method for delivering cell transplants for clinical translation. Nonetheless, the actions and cellular distribution of intravenously transplanted cells is a subject of controversy. Previous study showed that transplanted cells even survived for at least several weeks following intravenous transplantation of MSCs in animal models of SCI [27]. However, other studies reported that intravenously transplanted cells were primarily trapped in the lung, and secondarily in the spleen, liver, and kidney, with only a precious few cells found at the injured site [20, 28]. In the present study, we detected grafted cells in the lung, spleen, and injured spinal cord site, but found no such cells in the uninjured spinal cord after intravenous administration, which shows that the AD-MSCs can probably migrate into the injured spinal cord through the broken blood–spinal cord barrier.

The MSCs therapy was a result of indirect environmental modification rather than direct translineage conversion of migrated MSCs to functional oligodendrocytes or neurons [11]. Transplanted MSCs were able to reduce neurotoxicity and protect cells from apoptosis. MSCs secrete anti-apoptotic protein B-cell lymphoma 2 (Bcl-2), and prevent the release of Bcl-2-associated X protein and caspase 3, which are proapoptotic proteins [29, 30]. MSCs also act as neuroprotectors by secreting various angiogenic and neurotrophic factors such as brain-derived neurotrophic factor, nerve growth factor, vascular endothelial growth factor, and hepatocyte growth factor, thereby providing trophic support to damaged neurons [31]. In the present study, intravenous injection of AD-MSCs and/or MPSS increased levels of neuronal markers including β3-tubulin and GalC. This could be the result of endogenous neuronal cells that survived in the injured site through the protective effects of AD-MSCs. Furthermore, intravenously injected MSCs show peripheral immunoregulatory properties through inhibition of T-cell activities and through modulation of the host systemic and central nervous system inflammatory responses [32, 33]. It has been suggested that intravenously injected MSCs may not only be involved in systemic immune modulation, but may also migrate to the injured site to exert neuroprotective effects.

## Conclusion

In the current study, it is clear that the spinal cord underwent primary and secondary damages resulting from SCI. In this hostile environment, it is difficult to maintain cell survival, differentiation, and neuronal regeneration. Therefore, it is important to improve the environment by decreasing oxidative damage, LP, and inflammatory responses in order to protect intrinsic neural cells and recover their function. The MPSS treatment in SCI is controversial since it provides only modest neurological benefit despite the risk of serious adverse effects. Our results demonstrated that the intravenous injection of AD-MSCs in the acute spinal cord injured dog produced beneficial effects through enhancement of antioxidant and anti-inflammatory activities, and could be used as an alternative treatment modality in acute SCI.

## Abbreviations

AD-MSCs: Adipose-derived mesenchymal stem cells
Bcl-2: B-cell lymphoma 2
COX: Cyclooxygenase
DAPI: 4′,6-Diamidino-2-phenylindole
DMEM: Dulbecco’s modified Eagle’s medium
ELISA: Enzyme-linked immunosorbent assay
FBS: Fetal bovine serum
GalC: Galactosylceramidase
GFAP: Glial fibrillary acidic protein
GFP: Green fluorescent protein
H&E: Hematoxylin and eosin
4-HNE: 4-Hydroxynenonal
IL: Interleukin
LP: Lipid peroxidation
MAC: Minimum alveolar concentration
MPSS: Methylprednisolone sodium succinate
MSC: Mesenchymal stem cell
3-NT: 3-Nitrotyrosine
PBS: Phosphate-buffered saline
PC: Protein carbonyls
PN: Peroxynitrite
pSTAT3: Phosphorylated signal transducer and activator of transcription 3
SCI: Spinal cord injury
SD: Standard deviation
TBST: 10 mM Tris–HCl, pH 7.6, 150 mM NaCl, 0.05 % Tween-20
TNFα: Tumor necrosis factor alpha
